# High Energy Density Single-Crystal NMC/Li_6_PS_5_Cl Cathodes for All-Solid-State Lithium-Metal Batteries

**DOI:** 10.1021/acsami.1c07952

**Published:** 2021-07-29

**Authors:** Christopher Doerrer, Isaac Capone, Sudarshan Narayanan, Junliang Liu, Chris R. M. Grovenor, Mauro Pasta, Patrick S. Grant

**Affiliations:** † Department of Materials, University of Oxford, Oxford OX1 3PH, U.K.; ‡ The Faraday Institution, Quad One, Becquerel Avenue, Harwell Campus, Didcot OX11 0RA, U.K.

**Keywords:** solid-state battery, sulfide electrolyte, composite cathode, single-crystal NMC, interfacial contact, volume expansion, stack pressure, pressure dependence

## Abstract

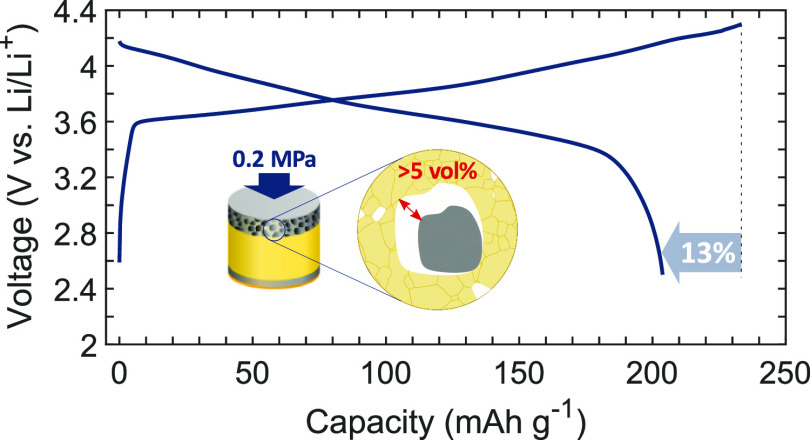

To match the high capacity of metallic anodes, all-solid-state batteries require high energy density, long-lasting composite cathodes such as Ni–Mn–Co (NMC)-based lithium oxides mixed with a solid-state electrolyte (SSE). However in practice, cathode capacity typically fades due to NMC cracking and increasing NMC/SSE interface debonding because of NMC pulverization, which is only partially mitigated by the application of a high cell pressure during cycling. Using smart processing protocols, we report a single-crystal particulate LiNi_0.83_Mn_0.06_Co_0.11_O_2_ and Li_6_PS_5_Cl SSE composite cathode with outstanding discharge capacity of 210 mA h g^–1^ at 30 °C. A first cycle coulombic efficiency of >85, and >99% thereafter, was achieved despite a 5.5% volume change during cycling. A near-practical discharge capacity at a high areal capacity of 8.7 mA h cm^–2^ was obtained using an asymmetric anode/cathode cycling pressure of only 2.5 MPa/0.2 MPa.

## 1 Introduction

An all-solid-state battery (ASSB) offers a safer alternative to conventional Li-ion batteries (LIBs) by avoiding the use of the flammable liquid electrolyte and has the potential to increase cell energy densities by ∼70% because the graphite anode is replaced with the Li metal.^1^ To balance the capacity of this metallic Li anode, a thick (50–200 μm) composite cathode comprising a mixture of an electrochemically active material and an inorganic solid-state electrolyte (SSE), such as a sulfide or oxide with high ionic conductivity (>1 mS cm^–1^), is required.^2^ A key challenge to ASSB performance is to achieve and maintain intimate interfacial contact and electrochemical stability between the particulate active material and the SSE within this composite cathode.^3^ Oxide-based SSEs generally have a wide electrochemical stability window but are hard and resistant to deformation; in contrast, sulfides have a relatively low flow stress even at room temperature (RT) and can be manipulated into intimate contact with the active particles by applying an external load during processing and/or service.^4^ Among the sulfides, Li_6_PS_5_Cl (LPSCl) is considered a promising candidate material because of its comparatively high ionic conductivity and ability to form a relatively stable passivation layer at the interface with Li.^5^

Lithium nickel manganese cobalt oxide (NMC) is a promising high energy density material for LIB cathodes with a discharge capacity >200 mA h g^–1^. However, when used in an ASSB, the NMC-based composite cathodes often show insufficient discharge capacity and/or are cycled at impractical stack pressures (≫5 MPa).^6,7^ Ruess et al. compared the cyclability of cathodes based on polycrystalline (PC) NMC in LIB and ASSB formats and observed NMC cracking during charge/discharge due to particle swelling/contraction and pulverization.^8^ This led to the progressive creation of new active surface that could be wetted by a liquid electrolyte but not by a SSE.^8^ Pulverization and the progressive loss of contact between the active material and the SSE results in rapid capacity reduction. Further, cracking may occur not only during cycling but during manufacture where pressures >100 MPa are frequently used in an effort to reduce or eliminate composite cathode porosity.^9^ Thus, there is a complex interplay between the pressures and strains experienced by composite cathodes during manufacture and service, and the integrity and resilience of the active/SSE interface, which in turn controls achievable capacity and cycle life.

In comparison with PC-NMC particles (an agglomeration of smaller crystalline particles), cathodes using single-crystal (SC) NMC particles (discrete, non-agglomerated crystallites) have shown relatively good cyclability in LIBs, ascribed to their more isotropic volume expansion during charge/discharge.^10,11^ Recently, SC-NMC-based cathodes have been investigated for ASSBs, albeit with relatively low Ni content to restrict cycling-induced volume changes (at the expense of reduced discharge capacity).^12−14^ Liu et al. investigated a SC LiNi_0.8_Mn_0.1_Co_0.1_O_2_/Li_10_SnP_2_S_12_/Li_4_Ti_5_O_12_ ASSB that delivered a maximum discharge capacity of 187 mA h g^–1^ under a stack pressure of 25 MPa.^15^ Although the cycling performance of the SC-NMC-based cathode was superior to a PC-NMC-based counterpart, the coulombic efficiency (CE) of the first cycle was only 74% and significantly lower than a conventional LIB equivalent (89%).

## 2 Results and Discussion

We contend that to achieve high energy ASSB composite cathodes, and to better realize the theoretical performance of the constituent materials, smart processing and cycling protocols are required that optimally engineer interaction between active particles, the SSE, and electron-conductive additives. The composite cathode fabrication route must provide percolating ionic and electronic networks and maximize the active/SSE contact area while minimizing porosity and damage. We demonstrate that SC LiNi_0.83_Mn_0.06_Co_0.11_O_2_/LPSCl-based cathodes with a high areal capacity (8.7 mA h cm^–2^) can provide both high discharge capacities (>200 mA h g^–1^) and high first cycle CE (>85%), even when cycled at pressures as low as 0.2 MPa.

**Figure 1:**
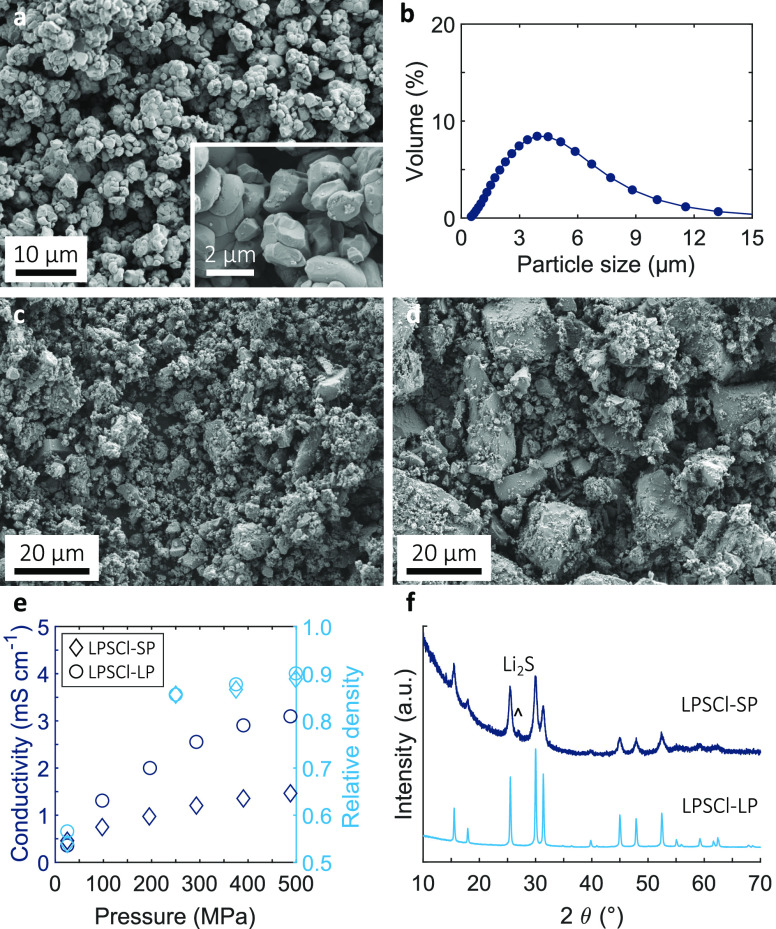
(a) SEM micrograph of the as-supplied SC-NMC powder and (b) corresponding particle size distribution. SEM micrograph of the (c) in-house LPSCl-SP used in the composite cathode and (d) as-supplied LPSCl-LP used in the SSE separator. (e) Ionic conductivity of LPSCl powders as a function of pressure, and (f) XRD spectra of the LPSCl powders.

Commercially available SC-NMC powders of small crystal size (1–2 μm) were chosen, as shown in the scanning electron microscopy (SEM) image in Figure 1a. The crystal structure and composition were confirmed by X-ray diffraction (XRD) and energy-dispersive X-ray (EDX) mapping, respectively, shown in Figures S1 and S2 (Tables S1 and S2). Electron backscatter diffraction (EBSD) images of cross-sectioned SC-NMC particles (Figure S3a) showed moderately agglomerated particles, with the particle size distribution obtained by dynamic light scattering (DLS) in Figure 1b and *D*_50_ = 3.4 μm. To restrict interfacial reaction with the LPSCl SSE, NMC powders were coated with LiNbO_3_ using a wet-chemical approach.^16,17^ EDX and X-ray photoelectron spectroscopy (XPS) analyses of coated SC-NMC particles shown in Figure S4 confirmed the presence of a Nb-rich surface oxide.

To understand any cycling benefits of the SC-NMC morphology, additional cells were prepared using chemically similar LiNi_0.8_Mn_0.1_Co_0.1_O_2_ powder but with PC-NMC morphology, using an identical processing methodology. Figure S3b shows an EBSD image from cross-sectioned PC-NMC particles, with significant agglomeration of secondary particles and *D*_50_ = 5.9 μm.

The particle diameter ratio between the active powder and SSE powder has an influence on subsequent mixing and consolidation behavior, and a ratio of ≥1 (SSE powder diameter ≤ active powder diameter) has been suggested to promote efficient active material utilization that is each active particle is intimately surrounded by SSE.^18^ Therefore, bespoke LPSCl powder was prepared to match the SC-NMC diameter by ball-milling a stoichiometric Li_2_S, P_2_S_5_, and LiCl mixture to give a relatively small particle (SP) size of 1–5 μm (Figure 1c). However, for the SSE separator and for convenience, we used commercial LPSCl, as shown in Figure 1d, with a relatively large particle (LP) size of 1–20 μm. LPSCl ionic conductivity was measured by electrochemical impedance spectroscopy with two stainless steel blocking electrodes at different stack pressures. Figure 1e shows a monotonic increase in ionic conductivity with relative density and stack pressure, for both SP and LP, up to 500 MPa, reaching 1.2 and 3 mS cm^–1^, respectively. Stack pressures >500 MPa increased impedance (Figure S5) due to excessive LPSCl particle cracking. The lower conductivity of LPSCl-SP may be explained by some unreacted Li_2_S suggested in the XRD spectra in Figure 1f. Both powders predominantly comprised the argyrodite phase. Carbon black (Super C65, 2.5 wt %) was used in all the cathodes as an electron conductivity aid.

**Figure 2:**
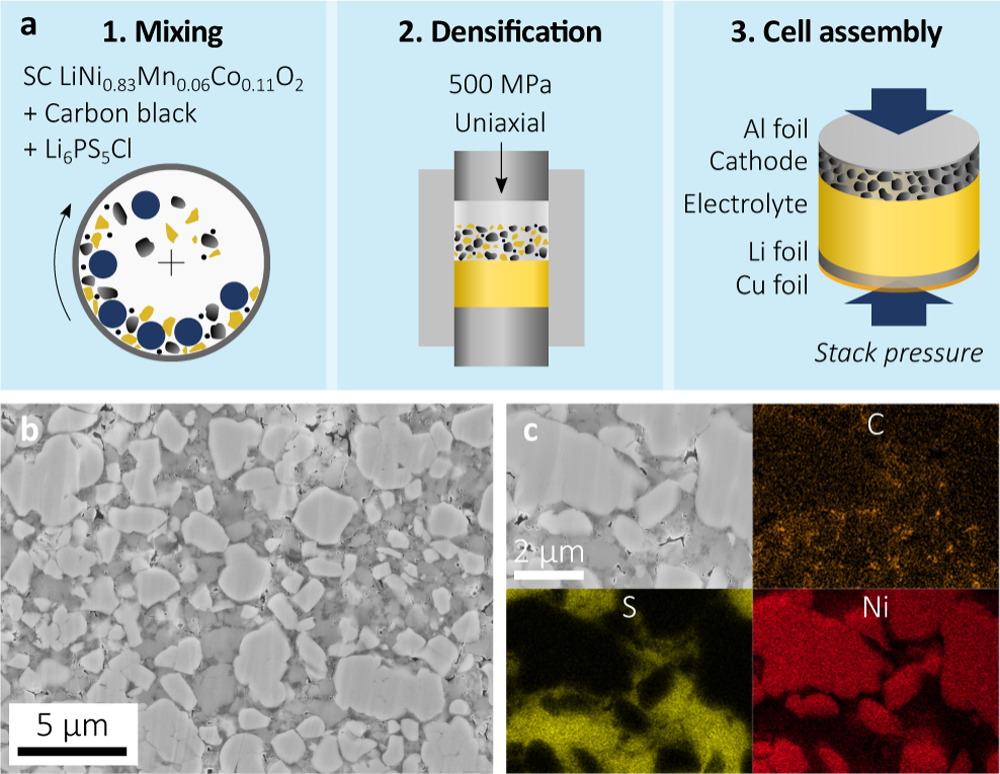
(a) Schematic of the cell manufacturing steps, (b) cross-section of a SC-NMC/LPSCl composite cathode after cell assembly and before cycling, and (c) EDX element maps for carbon, sulfur, and nickel.

Cells were assembled according to the steps shown schematically in Figure 2a. Active material, LPSCl-SP, and carbon black were mixed in a ball mill and subsequently pressed in a polymer mold at 500 MPa and RT with LPSCl-LP to form a pellet. Further details are available in Supporting Information. The SEM cross-section image in Figure 2b shows that the composite cathode exhibited no signs of the SC-NMC particle fracture despite the high loads used during mixing and pressing. Moreover, there was continuous, intimate contact at the SC-NMC/LPSCl interfaces and an overall relative density of ∼90%. Corresponding EDX element maps (Figure 2c) suggested an acceptable, typical distribution of carbon black to support electron percolation.

In contrast, densification of the similar PC-NMC/LPSCl mixture at the same pressure led to primary particle fracture along grain and/or agglomerate boundaries (Figure S6), with active fragments disconnected from one another and/or the LPSCl; the cracking persisted even when the fabrication pressure was lowered to 250 MPa, and only at 50 MPa was secondary particle integrity maintained. However, these lower pressures significantly increased porosity (from ∼14 to ∼22%, Figure S6) and were markedly less effective in promoting the NMC/LPSCl interfacial contact.

The different relative particle sizes of PC-NMC and SC-NMC affected the way they interacted with the LPSCl-SP during consolidation. As shown in Figure S7, relatively small LPSCl particles will tend to sit in the interstices between larger PC-NMC particles. On application of pressure, there is movement of the hard PC-NMC particles and shear forces are transmitted to the more easily deformed LPSCl. The movement and shearing of the softer LPSCl over the harder NMC surface promotes the required intimate interfacial contact. However, once the PC-NMC particles contact one another, relative motion is inhibited and it is difficult to induce any further shearing of the LPSCl, and the PC agglomerates may start to crack. For the smaller SC-NMC, where the particle diameters are more similar to LPSCl, greater relative motion and shear deformation of the LPSCl is allowed before SC-NMC hard particle contact. Consequently, the extent and quality of interfacial coverage of LPSCl over the available SC-NMC surface is increased.

Cell assembly was completed by adding a Li foil and a Cu current collector on the anode side and an Al current collector on the cathode side.

A cell stack pressure during cycling is recommended to inhibit void formation at the anode/electrolyte interface during Li plating and stripping.^19^ Therefore, cells were cycled in the custom-made cell, as shown in Figure S8a, with a uniaxial pressure set by a torque wrench, calibrated to the stack pressure using a load cell (Figure S8b). We chose a low stack pressure of 2.5 MPa to study the electro-chemo-mechanical effects of the SC-NMC/LPSCl and PC-NMC/LPSCl cathodes during the first charge/discharge cycle. Very low stack pressures, for example, 1 MPa at the anode tend to favor Li void formation and a high overpotential (at reasonable current densities of 0.2 mA cm^–2^); on the other hand, significantly higher pressures may promote particulate cracking and become impractical for larger scale cells or packs.^20^

**Figure 3:**
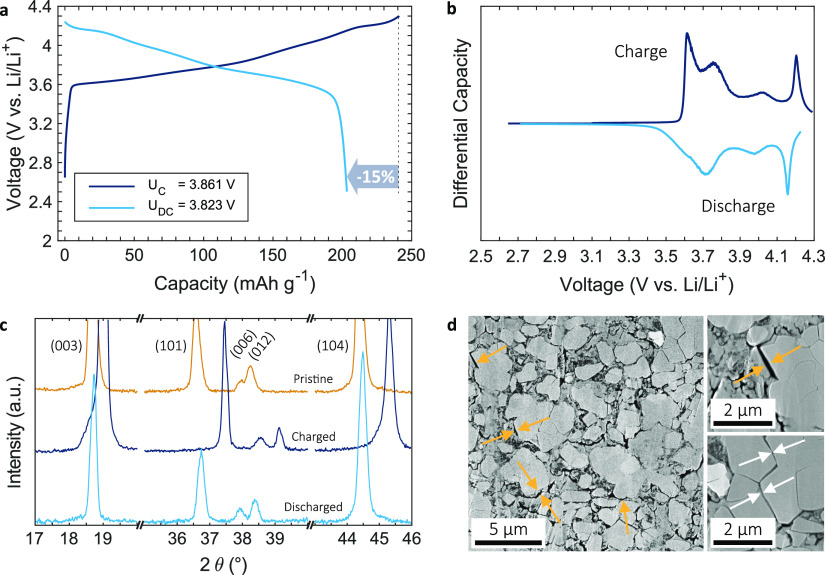
(a) Initial charge/discharge curves (with average voltages *U*_C_ and *U*_DC_) of a SC-NMC/LPSCl composite cathode (14 mg cm^–2^, 3 mA h cm^–2^) cycled at 2.5 MPa, 0.2 mA cm^–2^, 30 °C and the (b) differential capacity. (c) Ex situ XRD spectra before cycling, after charge and discharge. (d) Cathode cross-section after charging with some loss of contact at the SC-NMC/LPSCl interface and particle separation of previously agglomerated crystals.

Figure 3a shows the electrochemical performance of a SC-NMC/LPSCl composite cathode (14 mg cm^–2^, 3 mA h cm^–2^ areal capacity) during the first charge and discharge cycle at 2.5 MPa, 0.2 mA cm^–2^, and 30 °C. There was a high first cycle CE of 85% and a discharge capacity of 204 mA h g^–1^. The slope at the beginning of the charge (<3.6 V) was attributed to carbon/LPSCl redox activity that undermined first cycle CE, forming thermodynamically more stable decomposition products such as P_2_S_*x*_, LiCl, and S that then act as a passivation layer over subsequent cycles;^21−24^ cells showed CE > 99% on the second and subsequent cycle. For comparison, PC-NMC/LPSCl cathodes shown in Figure S9a had a first discharge capacity of only 152 mA h g^–1^ and a first cycle CE of ∼70% under the same cycling conditions. The PC-NMC/LPSCl cathodes also had a higher overpotential due to their higher microstructural heterogeneity and defect density. Further, the interfacial resistance of the SC-NMC cell (∼40 Ω cm^2^) was about half that of its PC-NMC counterpart (Figure S10) and was among the lowest reported for cells using a RT-pressed sulfide/NMC cathode and a Li anode.

Differential capacity curves (Figure 3b) showed characteristic redox peaks previously observed for high-Ni NMC in LIBs, which indicated multiphase structural transitions.^25^ A sharp peak at ∼4.2 V, rarely observed in lower Ni NMC materials, but typical at relatively high Ni contents, related to the appearance of the H3 phase and a more marked volume change.^26^ From ex situ XRD before cycling, after charge and after discharge (Figure 3c), the lattice volumes before and after charging were estimated at 101.62 and 96.08 Å^3^, respectively, corresponding to a near reversible volume shrinkage of 5.5% and consistent with the literature.^26^

Corresponding cross-section SEM images after charging in Figure 3d showed instances of contact loss at the SC-NMC/LPSCl interface (orange arrows) and particle separation of previously agglomerated crystals (white arrows) due to anisotropic volume change. There was no fracture of non-agglomerated SC-NMC particles. Similar images of PC-NMC/LPSCl cathodes (Figure S9b) after the first charge showed interfacial contact loss and intergranular cracks. There was a notable tendency for larger diameter primary particles to undergo intergranular cracking and dis-aggregation of secondary particles because of their more restricted ability to accommodate cycling strains without debonding.^27^

Based on the qualitative insights described above, Figure S11 shows a schematic depiction of the different changes in microstructure for SC-NMC- and PC-NMC-based composite cathodes during manufacturing and then cycling.

To study the effect of the intrinsic volume changes of the NMC and any interfacial contact loss with the SSE during cycling, the cyclability of the SC-NMC/LPSCl cathodes was investigated at different stack pressures (50, 10, 2.5, and 0.2 MPa). Figure S12a compares the first cycle CE at different stack pressures. At 50 MPa, there was cell failure on the first charge (Figure S12b) with a qualitatively similar charging curve to that shown by Doux et al. at a stack pressure of 25 MPa, which was ascribed to accelerated Li penetration across the SSE separator.^28,29^ Cells at lower pressures of 10 and 2.5 MPa showed an almost identical first cycle CE of ∼85% and then failed at higher cycles (∼10) presumably via a similar mechanism.

**Figure 4:**
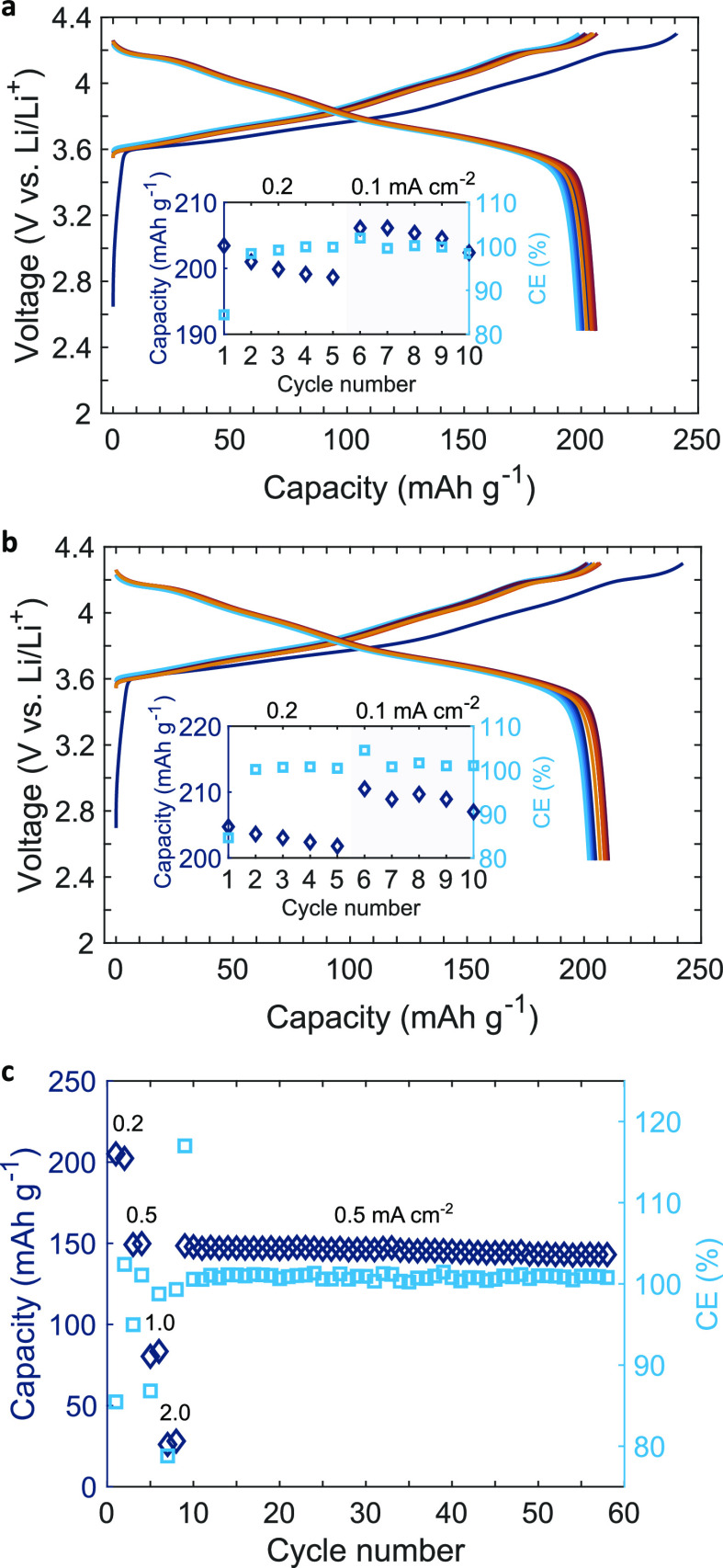
Charge/discharge curves of a SC-NMC/LPSCl composite cathode (14 mg cm^–2^, 3 mA h cm^–2^) with a Li anode, and capacity fade (inset) at (a) 2.5 and (b) 10 MPa. (c) Discharge capacity at different current densities and higher cycle numbers of the same SC-NMC/LPSCl cathode with a LTO/LPSCl composite anode cycled at 10 MPa.

Figure 4a,b shows in detail the charge and discharge curves of the first 10 cycles of 14 mg cm^–2^ SC-NMC/LPSCl composite cathodes at 0.2 mA cm^–2^ and at stack pressures of 2.5 and 10 MPa, respectively. The initial discharge capacities were 204 and 205 mA h g^–1^ and, as shown in the inset graphs, the capacity retention after 10 cycles was 99.4 and 101.1% (>100% was possible because the current density was lowered to 0.1 mA cm^–2^ after 5 cycles) for 2.5 and 10 MPa, respectively. At a lower pressure of 2.5 MPa, progressive NMC/LPSCl contact losses were more likely to occur on each cycle. However, at 10 MPa, even after 10 cycles, cells had a high discharge capacity (∼210 mA h g^–1^), which is amongst the highest reported for RT-pressed NMC/sulfide cathodes in ASSBs. The sensitivity of capacity after five cycles to current density indicated that electrochemical processes were time dependent, and that further optimization will be required to enable faster charging/discharging, for example, further optimization of the cathode microstructure or an electrolyte with higher intrinsic ionic conductivity.^30^

To explore even higher cycle numbers for the SC-NMC/LPSCl cathode, we replaced the Li anode with a Li_4_Ti_5_O_12_ (LTO)/LPSCl composite anode to avoid the problem of Li penetration and premature failure; LTO also benefits from a relatively small volume change (0.2%) on charge/discharge.^14^ In this arrangement, Figure 4c shows that the same SC-NMC/LPSCl cathodes had a capacity retention of 96.3% after 50 cycles (between cycle number 9 and 58). Figure S9c shows that the PC-NMC/LPSCl cathodes again had lower discharge capacities that decayed faster (92.4% after 50 cycles), due to their higher defect density and accelerated particle pulverization.^15^

**Figure 5:**
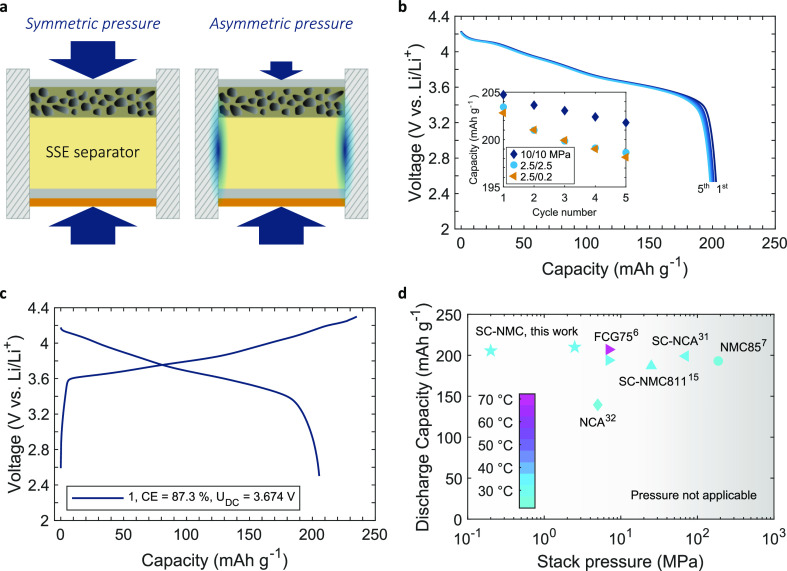
(a) Asymmetric load cell setup used to lower the cathode stack pressure while maintaining anode pressure. Corresponding voltage capacity curves for a cell cycled at 0.2 mA cm^–2^ and an anode/cathode pressure of 2.5 MPa/0.2 MPa for a (b) SC-NMC/LPSCl composite cathode of 14 mg cm^–2^ (3 mA h cm^–2^), and capacity fade compared to different pressures and a (c) SC-NMC/LPSCl composite cathode of 43 mg cm^–2^ (8.7 mA h cm^–2^). (d) Discharge capacity comparison for Ni-rich composite cathodes pressed at RT as a function of the stack pressure during cycling.

To explore further the effects of a low stack pressure at the cathode, a new asymmetric pressure design, as shown schematically in Figure 5a, was implemented. Whereas a compression spring (Figure S8c and Table S3) was used to apply 0.2 MPa at the cathode, the pressure on the anode was maintained at 2.5 MPa using a calibrated uniaxial load. Pressure asymmetry was possible because of the high friction coefficient and contact area between the SSE pellet and the mold wall. Cathode and anode pressures were confirmed experimentally using a load cell and computationally by finite element method calculations that showed under reasonable friction assumptions, when the anode was loaded to 2.5 MPa, there was negligible pressure transmission to the cathode (Figure S13). Figure S12a shows that cells cycled at a cathode pressure as low as 0.2 MPa only had a slightly reduced first cycle CE of ∼83% compared with those at higher pressure in Figure 4. The first five discharge cycles at 2.5 MPa/0.2 MPa are shown in Figure 5b for a 14 mg cm^–2^ cathode at 0.2 mA cm^–2^, and the inset shows the discharge capacity at different pressure combinations. While the slowest decay in capacity was for a 10 MPa/10 MPa arrangement as previously suggested in Figure 4b, the discharge capacity of the first cycle and the capacity decay over the first five cycles was almost identical for 2.5 MPa/2.5 MPa and 2.5 MPa/0.2 MPa arrangements.

Cycling of a SC-NMC composite cathode with an increased, very high areal capacity of 8.7 mA h cm^–2^ (thickness ∼200 μm) was then demonstrated at a cathode pressure of 0.2 MPa in Figure 5c, providing a discharge capacity of 205 mA h g^–1^ and a first cycle CE of 87%. To understand how this performance might scale toward a practical cell, a SSE separator thickness reduced from 1.5 mm to 20 μm was assumed, and including the mass/volume of the Li foil and current collectors, (see Table S4 for details), a cell based on this arrangement was estimated to provide a specific energy of 405 W h kg^–1^ and a volumetric energy density of 1069 W h L^–1^.

A comparison of discharge capacities obtained in this work with other RT-pressed cathodes as a function of applied stack pressures during cycling is shown in Figure 5d.^31,32^ Our results suggest that by (i) choosing active and SSE particle sizes and morphologies matched to their required function, (ii) the use of protective coatings, and (iii) optimizing mixing and consolidation parameters, high initial capacity and high efficiency can be achieved even at low stack pressures, including novel low-pressure asymmetric arrangements.

## 3 Conclusions

We have demonstrated that cold-pressed SC NMC/LPSCl cathodes can deliver high first cycle CE >85% and discharge capacities >205 mA h g^–1^ even when cycled at a low, practical external stack pressure 0.2 MPa and despite the relatively high volume expansion/contraction of the NMC with relatively high Ni content. Critical to the performance was an optimized fabrication route that maximized NMC/LPSCl physical surface interaction and interfacial contact. SC NMC particles showed greater robustness during mixing and densification in comparison with their PC NMC counterparts that suffered from intergranular cracks first during processing and then during cycling due to anisotropic volume expansion. The optimized SC NMC/LPSCl composite cathode microstructure facilitated a novel anode/cathode asymmetric pressure arrangement that allowed pressures as low as 2.5 MPa/0.2 MPa to be used successfully.

## Supporting Information Available

Experimental details, XRD spectra and EDX composition analysis of pristine SC-NMC, Rietveld refinement results, EBSD images of SC-NMC and PC-NMC particles, surface analysis of LiNbO_3_-coated SC-NMC particles using EDX and XPS, Nyquist plots of LPSCl-SP measured at different pressures, SEM cross-sections of PC-NMC/LPSCl cathodes densified at different fabrication pressures, schematic comparison between SC/PC-NMC/LPSCl cathodes during densification, schematic cell setup, initial charge/discharge curves of a PC-NMC/LPSCl cathode including SEM cross-section after charging, discharge capacity decay of a PC-NMC/LPSCl/LTO cell, schematic of PC/SC particles during manufacturing and cycling, Nyquist plot of charged cells, CE at different stack pressures and corresponding charge curves, finite element analysis of a pellet with asymmetric pressure, and details of the energy density calculation (PDF)
