# Supplementary material for: High Energy Density Single-Crystal NMC/Li6PS5Cl Cathodes for All-Solid-State Lithium-Metal Batteries
Source: ACS Appl Mater Interfaces. 2021 Jul 29;13(31):37809–15. doi: 10.1021/acsami.1c07952 (PMC8397257; doi:10.1021/acsami.1c07952)
Supplement: Supplementary file 1 — am1c07952_si_001.pdf [file am1c07952_si_001.pdf]

Supporting Information:

High Energy Density Single Crystal NMC/Li_6PS_5Cl Cathodes for All-Solid-State Lithium Metal Batteries

Christopher Doerrer,[*,†] Isaac Capone,[†] Sudarshan Narayanan,[†] Junliang Liu,[†] Chris R. M. Grovenor,[†,‡] Mauro Pasta,[†,‡] and Patrick S. Grant[*,†,‡]

†*Department of Materials, University of Oxford, Oxford OX1 3PH, UK*

‡*The Faraday Institution, Quad One, Becquerel Ave, Harwell Campus, Didcot OX11 0RA, UK*

E-mail: christopher.doerrer@materials.ox.ac.uk; patrick.grant@materials.ox.ac.uk

Experimental/Methods

Dynamic light scattering (DLS). The particle size distribution was measured at room temperature using DLS in a Horiba LA-960 analyzer operating with a 650 nm red laser and a 450 nm blue light emitting diode (LED). The particles were first dispersed in deionized water using a bath sonicator for 1 min.

Electrode preparation. In-house Li_6PS_5Cl (LPSCl-SP) was made by mixing a stoichiometric ratio of Li_2S, P_2S_5 and LiCl in a 40 ml grinding bowl containing 16 × 10 mm zirconia balls in a planetary mill (Fritsch Pulverisette 7) at 600 rpm for 14 h. NMC particles were coated with $LiNbO_3$ based on a wet-chemical approach using lithium niobium ethoxide solution (Alfa Aesar, 5% w/v in ethanol). 1 g NMC was stirred in 400 µL lithium niobium ethoxide solution and 800 µL anhydrous ethanol for 1 h. The solvent was then evaporated under vacuum at 50 °C before the sample was heated under air to 350 °C (heating/cooling rate: 10 °C min^{-1}, holding temperature: 30 min) to calcine the cathode particles. For the composite cathode, 70 wt% $LiNbO_3$ coated NMC, 27.5 wt% LPSCl-SP and 2.5 wt% carbon black (Super C65) was mixed in a planetary ball mill (Fritsch Pulverisette 7) at 140 rpm for 30 min. $Li_4Ti_5O_{12}$ (LTO)/LPSCl anodes were prepared using the same mixing conditions comprising 60 wt% LTO, 35 wt% LPSCl and 5 wt% carbon nanofibers.

Cell Assembly. Cells were assembled and cycled in a custom-designed device involving a polyether ether ketone (PEEK) mold and two metallic plungers. Stacked layers of 25 mg (50 mg for cells cycled at asymmetric pressure) of LPSCl-LP and then 4 mg (12 mg for the cell with a high loading in Figure 5c) of the composite cathode mixture were pressed in a PEEK mold using a WC die set at 500 MPa. A Li foil of ∼30 µm thickness or 15 mg of the composite anode mixture was added to the stacked cell as anode. All work was carried out in an Ar-filled glovebox (O_2 and H_2O levels <1 ppm).

Electrochemical measurements. Electrochemical impedance spectroscopy (EIS) was performed in a Gamry Instruments Interface-1000 potentiostat using a voltage amplitude of

10 mV in the frequency range from 1.6 MHz to 0.1 Hz. Galvanostatic cycling was performed at a constant current (0.1 or 0.2 mA cm^{-2}) between 4.3 V and 2.5 V at 30 °C.

X-ray diffraction (XRD). XRD measurements were carried out in a Rigaku MiniFlex with Cu $K\alpha_1$ radiation inside an Ar filled glovebox. Patterns were collected from 10° to 90° in $\theta/2\theta$ using a step size of 0.01° at RT. Rietveld refinements were implemented in Profex software.

Scanning electron microscopy (SEM) and energy-dispersive X-ray spectroscopy (EDX). Sulfide-based pellets were cross-sectioned by an automated Ar ion polishing system (PECS 2, Gatan) and transferred under vacuum into a Zeiss Merlin scanning electron microscope using an airtight transfer device. Images were taken by the Everhart Thornley and in lens secondary electron detectors. EDX was carried out by an Oxford Instruments X-Max 150 silicon drift detector and analysis was conducted in AZtec software.

Electron backscatter diffraction (EBSD). Samples were prepared by coating NMC particles (1 g) dispersed in a polyvinylidene fluoride (PVDF)/N-Methyl-2-pyrrolidone (NMP) solution (50 mg/1 ml) onto an Al foil to obtain electrode sheets which were subsequently cross-sectioned by an automated Ar ion polishing system (PECS 2, Gatan). EBSD characterization was carried out in a Zeiss Merlin FEG-SEM system equipped with a Bruker e-flash high resolution EBSD detector. An accelerating voltage of 30 kV and a probe current of 10 nA was used to acquire Kikuchi patterns with resolution 400 × 300 at a step size of 50 nm. Data was expressed as band contrast (BC) and inverse pole figure (IPF). The lattice parameters of pristine materials shown in Table S1 were used for EBSD pattern indexing.

Finite Element Method (FEM) stress analysis. Geometries and calculations were implemented with Inventor software. It was assumed that the pellet was fixed at the outer surface due to its high friction with the plastic mold.

X-ray photoemission spectroscopy (XPS). XPS was conducted using a PHI VersaProbe

III system generating monochromatic *Al* X-rays at 1486 eV. $LiNbO_3$-coated NMC powder was mounted on adhesive carbon tape inside a glovebox and transferred into the XPS via a vacuum transfer vessel to avoid contamination and ambient exposure. Survey scans and high-resolution elemental scans were acquired at pass energies of 224 eV and 55 eV respectively. The powder was further etched using a monoatomic Ar^+ ion beam accelerated at 1 kV and rastered over a 3×3 mm^2 scan area for a total of 40 min, while profiling XPS spectra in 2 min (15 min) and 5 min (25 min) time intervals. The spectra were fitted to Gaussian-Lorentzian and Voigt lineshapes (where asymmetry was pronounced), and quantified using CasaXPS software.

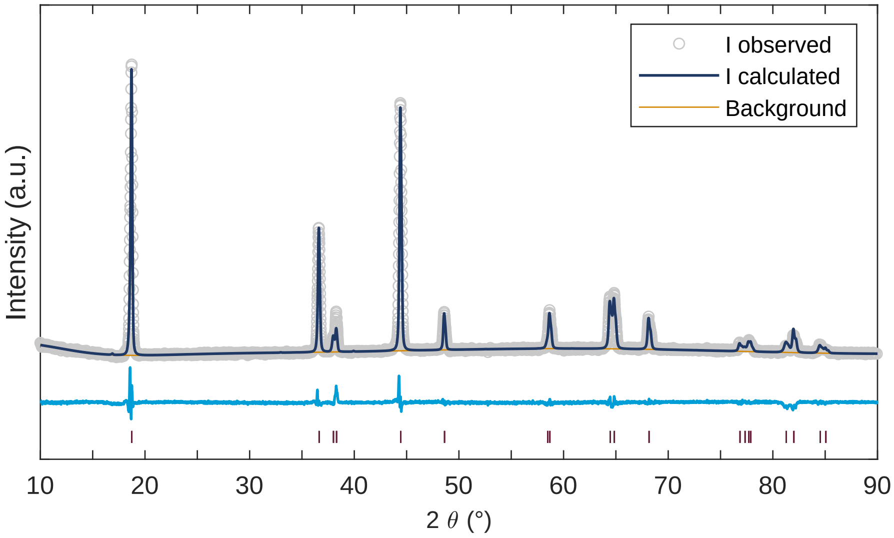

Figure S1: XRD spectra of pristine SC-NMC and corresponding Rietveld refinement.

Table S1: Rietveld refinement of SC-NMC using R$\bar{3}$m space group.

	Pristine	Charged (4.3 V)	Discharged (2.5 V)
a (Å)	2.8727	2.81354	2.8686
c (Å)	14.1958	14.0151	14.2336
χ^2	2.04	3.96	1.08
R_{exp}	1.47	1.86	2.4
GoF	1.43	1.99	1.04

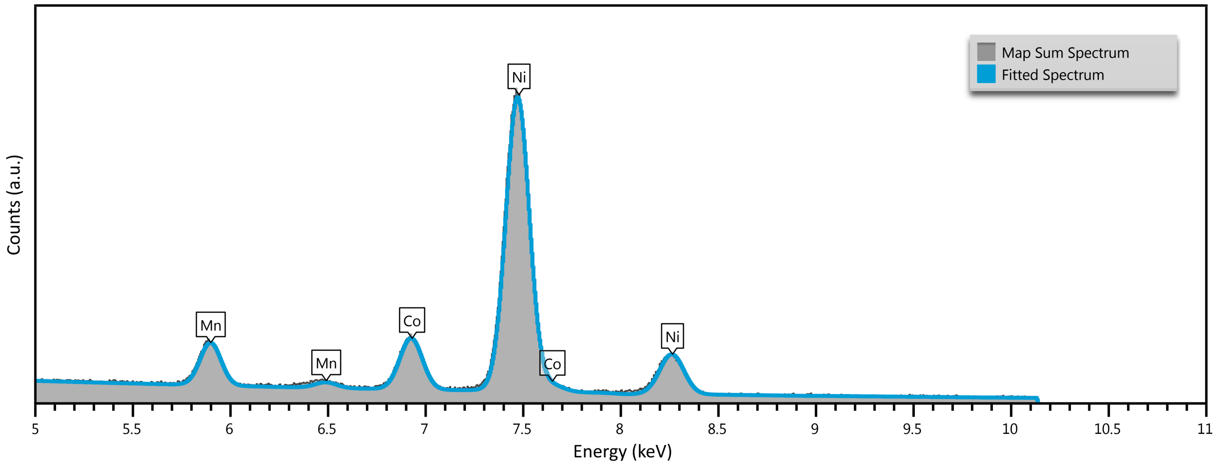

Figure S2: EDX spectra of pristine SC-NMC particles.

Table S2: Composition of SC-NMC from the EDX analysis shown in Figure S2.

Element	Nominal (%)	Measured (%)
Ni	83	82.8
Co	11	11.3
Mn	6	5.9

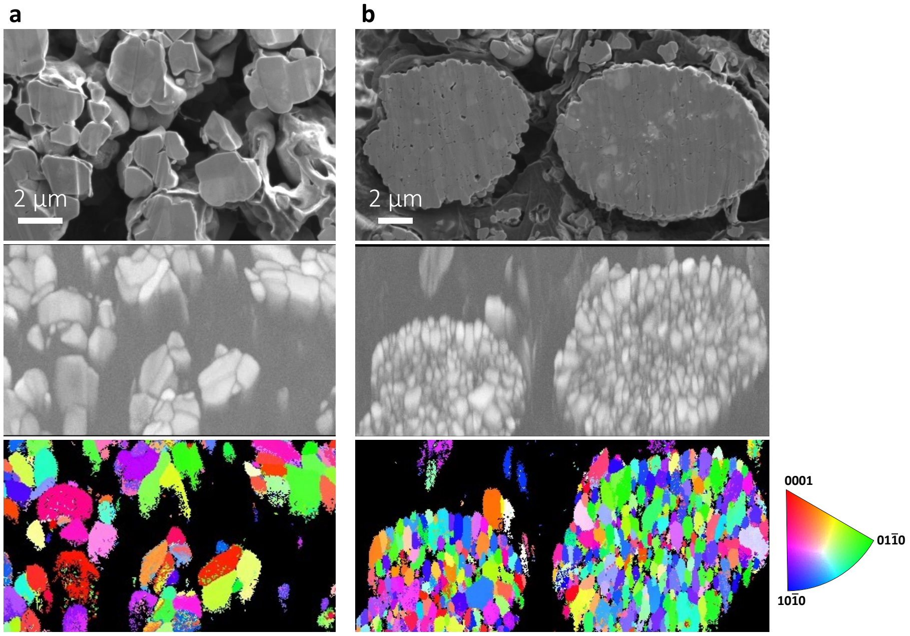

Figure S3: SEM image, pattern quality map and false colored grain map of (a) SC-NMC and (b) PC-NMC particles.

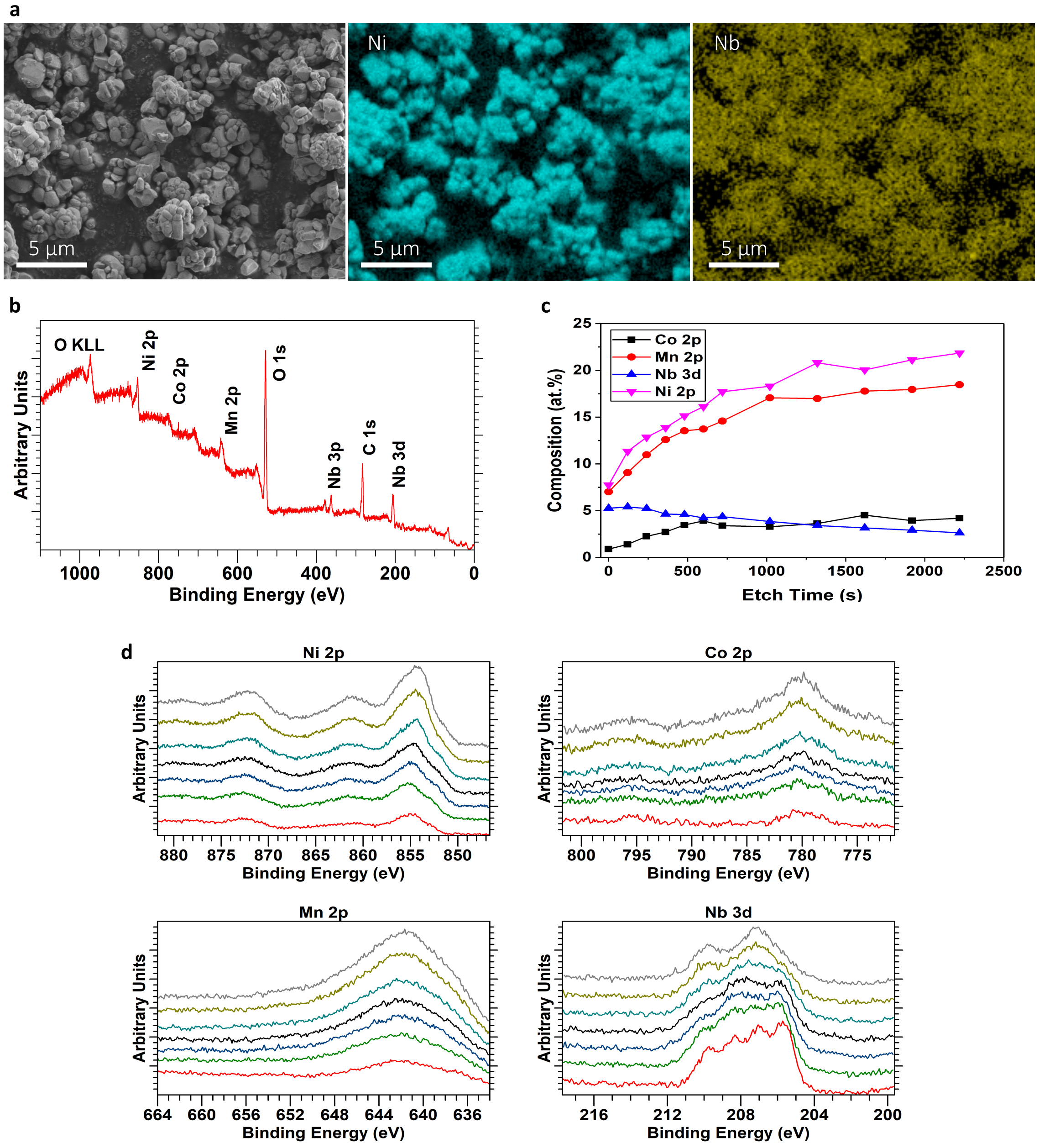

Figure S4: (a) SEM images of $LiNbO_3$-coated SC-NMC particles and corresponding EDX element maps for Ni and Nb. (b) XPS survey spectra for $LiNbO_3$-coated SC-NMC powder indicating characteristic peaks for Ni, Mn, Co, Nb and O; (c) estimated composition depth profile from XPS quantified high-resolution spectra as a function of etch time; and (d) corresponding XPS spectra stacked in order of increasing etch time.

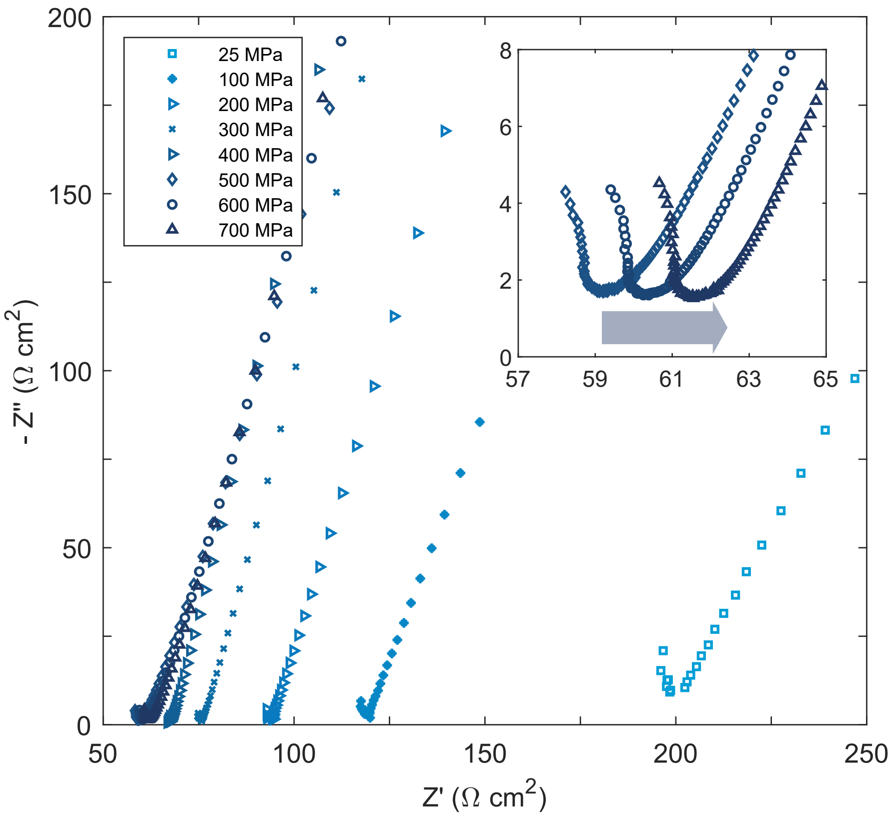

Figure S5: Nyquist plots of LPSCl-SP at different pressures (all at RT) as a function of pressure up to 700 MPa. The arrow in the inset emphasizes that the resistance slightly increased after 500 MPa.

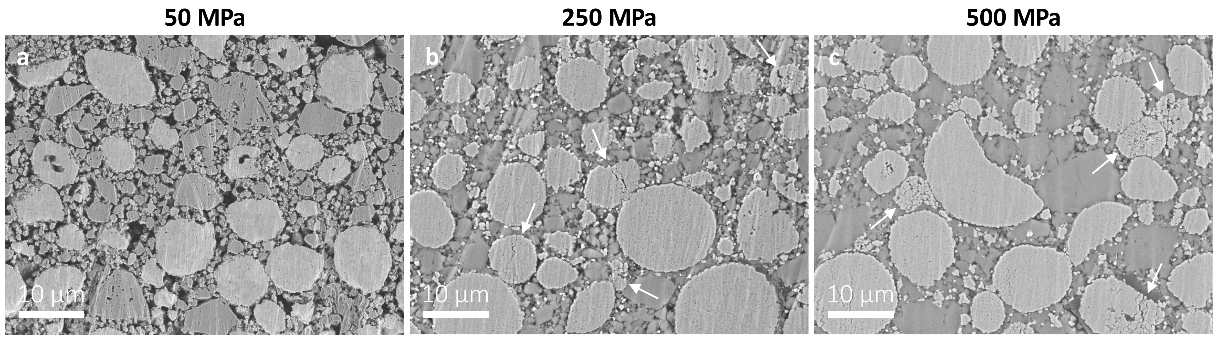

Figure S6: PC-NMC/LPSCl cathodes densified at different fabrication pressures. Particle fracture is indicated by the white arrows. Fabrication pressures and estimated relative densities were: (a) 50 MPa, 78% (b) 250 MPa, 82% (c) 500 MPa, 86%.

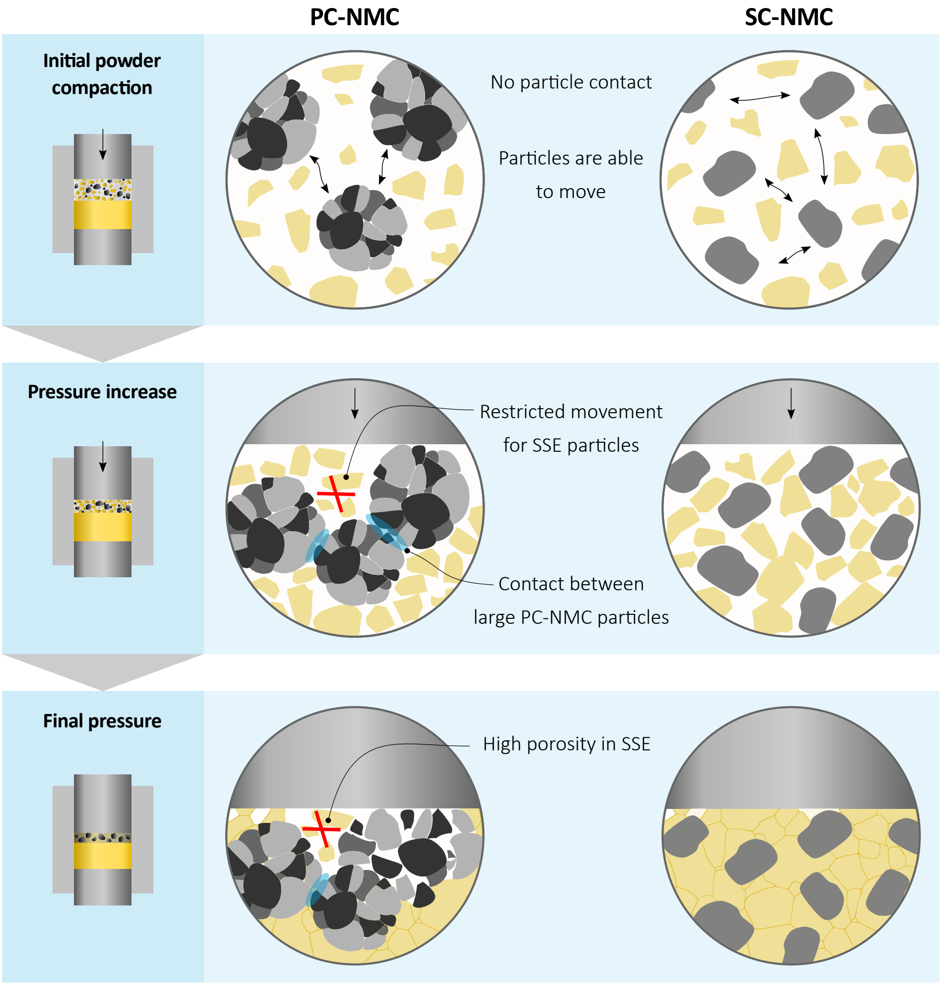

Figure S7: Schematic comparison between polycrystalline (PC-NMC, grey/black agglomerates) and single-crystalline (SC-NMC, grey particles) cathode particles and solid-state electrolyte (SSE, yellow) interactions during densification.

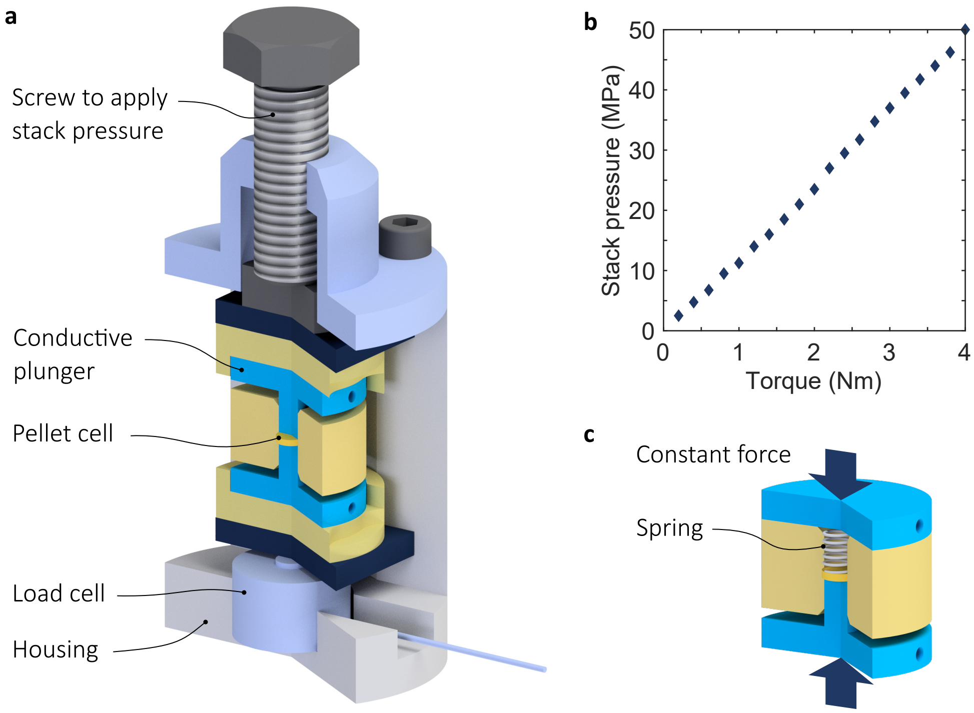

Figure S8: (a) Set-up and design used to apply a symmetric stack pressure at the start of cycling. The screw shown on top is centred with the pellet which allows fine adjustment of a uniaxial pressure. (b) Stack pressure as a function of torque determined by a load cell for a pellet with a diameter of 5 mm. (c) Set-up used to apply an asymmetric stack pressure by means of a compression spring. The force from the spring measured by a load cell was 4 N which results in a cathode load of 0.2 MPa and compares well to the calculated values in Table S3.

Table S3: Details of the compression spring used in an asymmetric pressure set-up and resulting load on the cathode. Calculations are based on the analytical equations summarized by Stewart.[S1]

Shear modulus (MPa)	81500
Wire thickness (mm)	0.4
Mean diameter (mm)	4.15
Active coils	5
Spring constant (N/mm)	0.73
Spring deflection (mm)	5
Force (N)	3.65
Cell area (mm^2)	19.63
Cathode load (MPa)	0.19

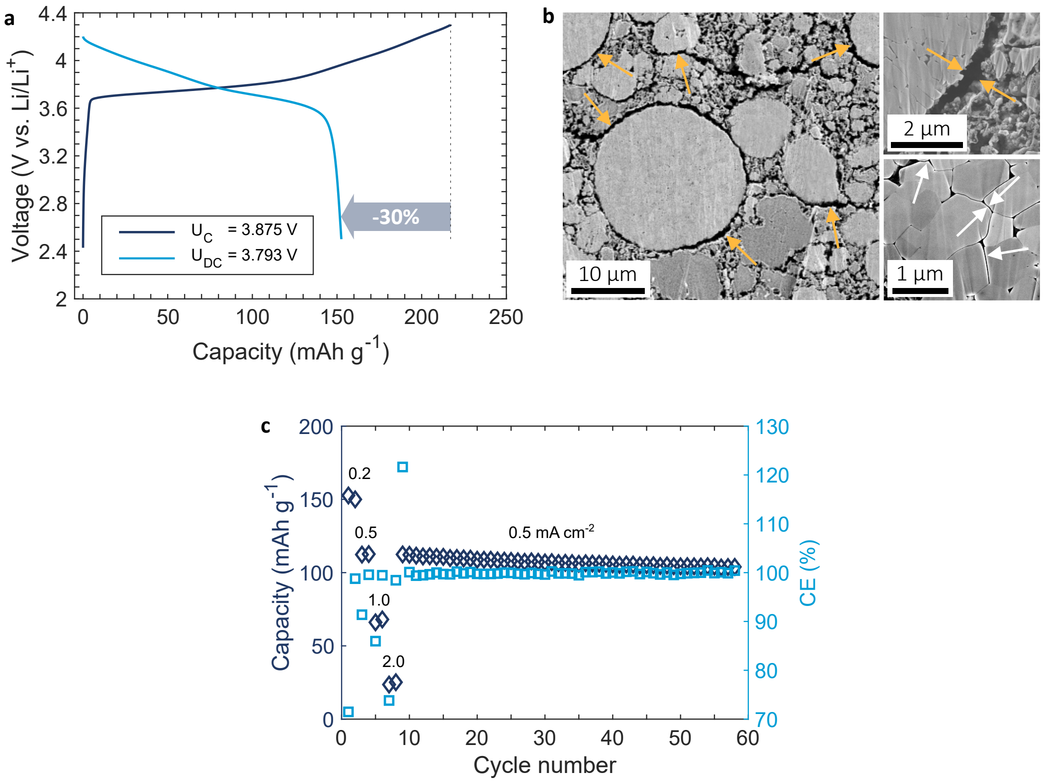

Figure S9: (a) Initial charge/discharge curves of a PC-NMC/LPSCl cathode with a loading of 14 mg cm^{-2} (2.2 mAh cm^{-2}) and a Li anode cycled at 2.5 MPa, 0.2 mA cm^{-2}, 30 °C. (b) Cross-section of a PC-NMC/LPSCl composite cathode after charge. (c) Cyclability of a PC-NMC/LPSCl/LTO cell with the same cathode loading at 10 MPa.

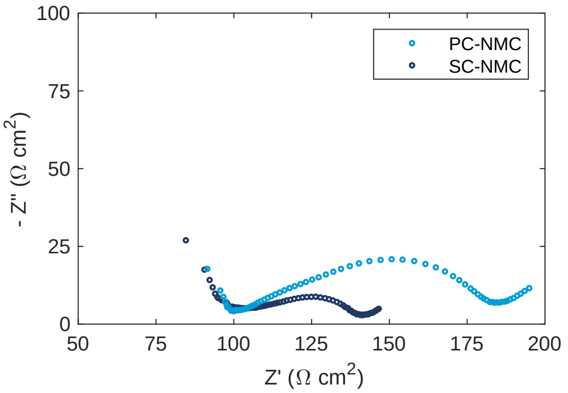

Figure S10: Comparison of the interfacial resistance for composite cathodes based on PC-NMC/LPSCl and SC-NMC/LPSCl. Measurements were taken after the cells were charged to 4.3 V (10 MPa, 30 °C).

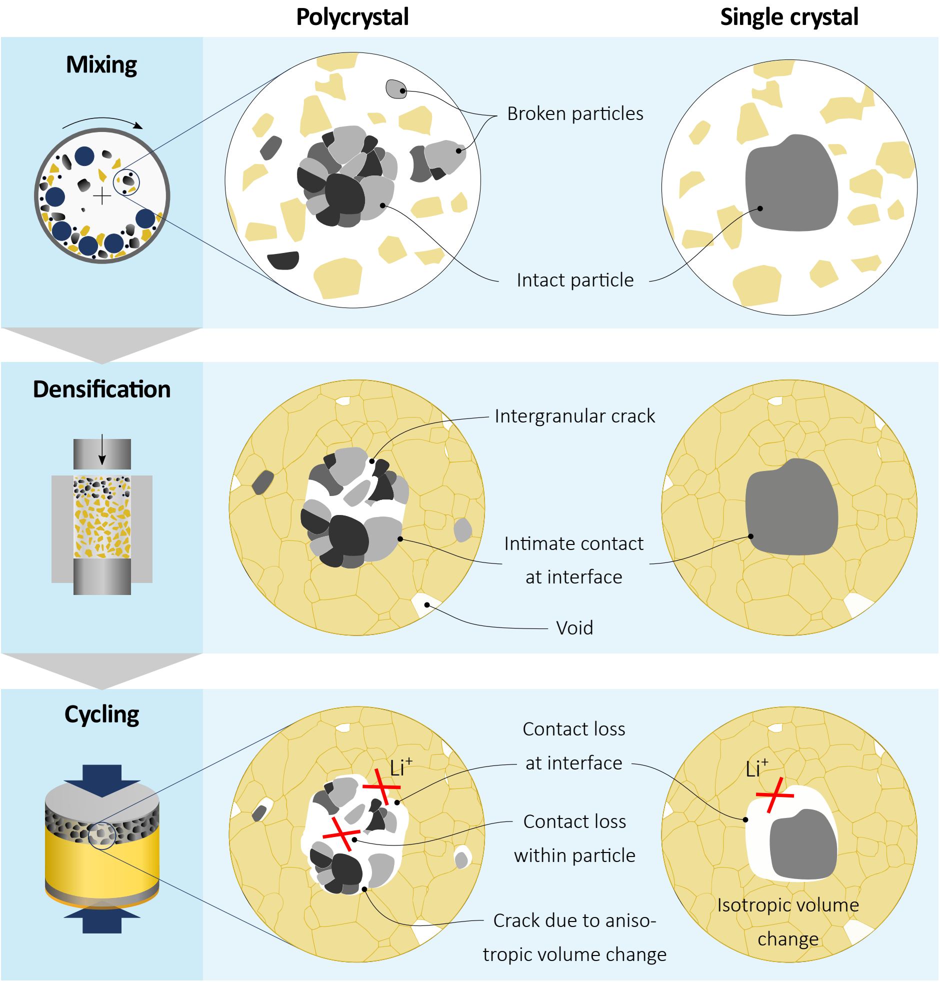

Figure S11: Schematic comparison between polycrystalline (PC-NMC) and single-crystalline (SC-NMC) composite cathode particles during manufacturing and cycling.

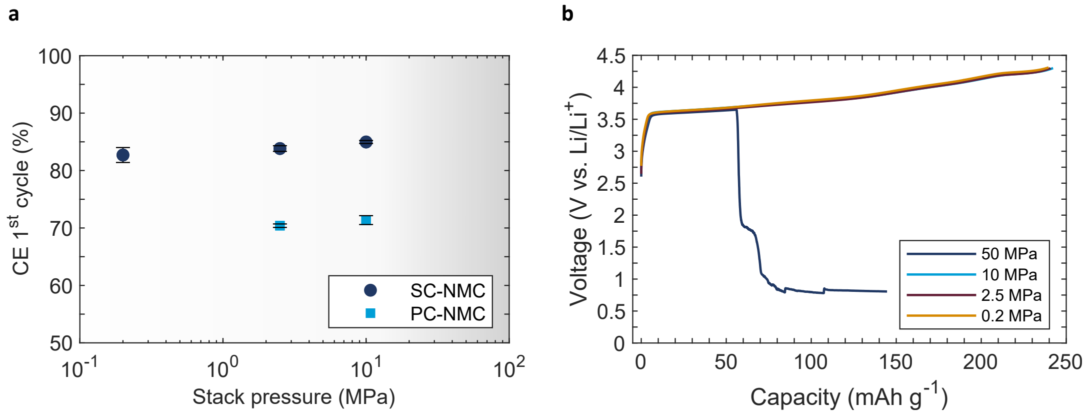

Figure S12: (a) First cycle coulombic efficiency (CE) of a SC-NMC/LPSCl composite cathode obtained at different stack pressures. (b) Comparison of charge curves of SC-NMC/LPSCl composite cathodes cycled at different stack pressures. The cell at 0.2 MPa was cycled at an asymmetric pressure with 2.5 MPa at the anode.

Table S4: Parameters of the SC-NMC/LPSCl/Li cell with a high cathode loading used for energy density calculations. All values were those used/obtained experimentally except the separator thickness which was 1.5 mm in the actual experiment and assumed to be 20 µm in the calculations.

Cathode	NMC loading (mg cm^{-2})	42.42
	NMC ratio (wt%)	70
	LPSCl ratio (wt%)	27.5
	Carbon black ratio (wt%)	2.5
	Relative density	0.9
	Al current collector thickness (µm)	15
Anode	Li foil thickness (µm)	50
	Cu current collector thickness (µm)	10
Separator	Thickness (µm)	20
	Relative density	0.9
Cell	Discharge capacity (mAh g^{-1})	205.5
	Average discharge voltage (V)	3.674
	Cell diameter (mm)	5
Cycle conditions	Cathode stack pressure (MPa)	0.2
	Anode stack pressure (MPa)	2.5
	Current density (mA cm^{-2})	0.2

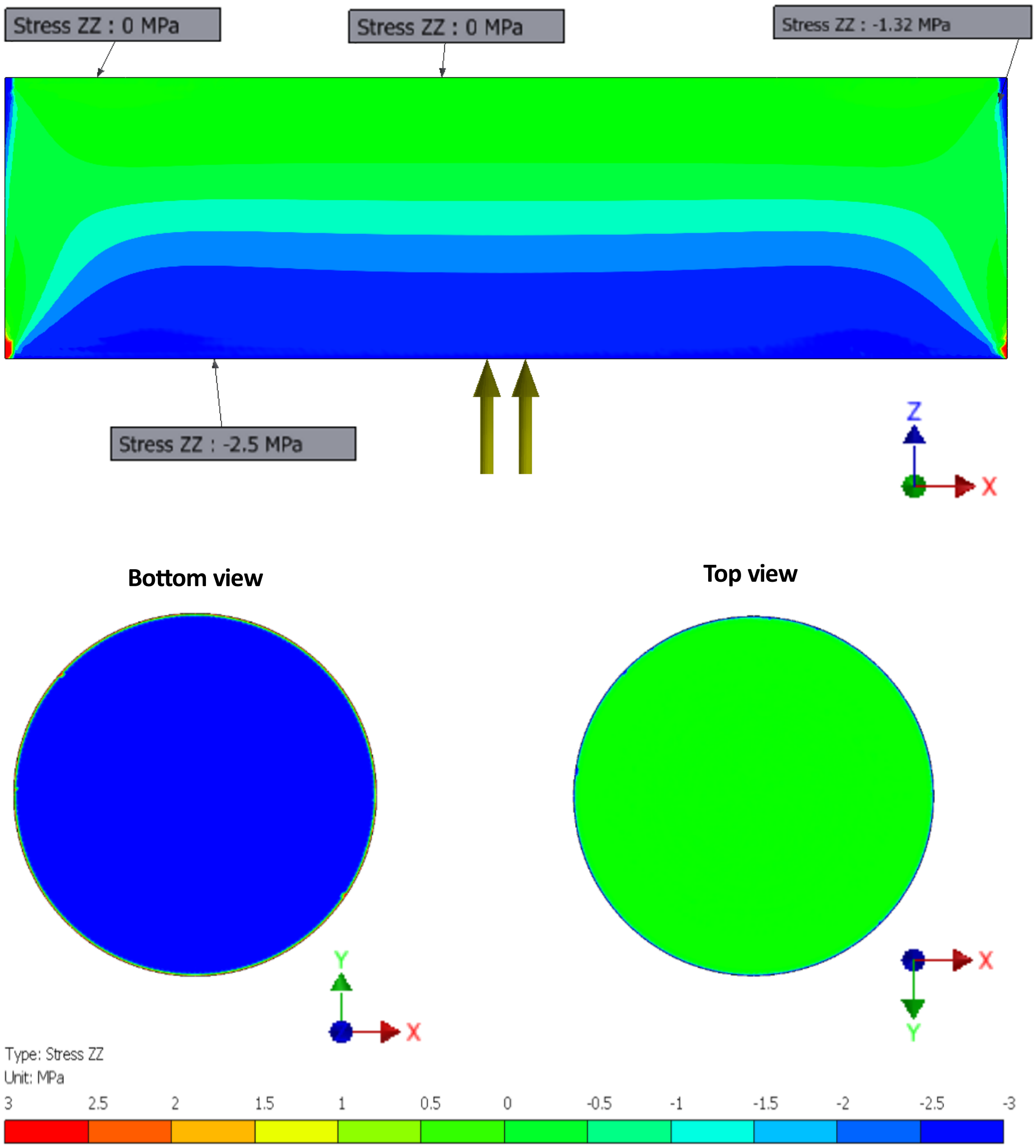

Figure S13: Finite element analysis of the pellet with a one-sided (asymmetric) stack pressure applied showing negligible pressure (stress) transmitted from the anode to the cathode due to wall friction. The bottom represents the anode where a loading of 2.5 MPa was applied. The top view shows that there was negligible stress induced in the z direction in the cathode.

References

(S1) Stewart, M. L. A Computational Approach for Evaluating Helical Compression Springs. *International Journal of Research in Engineering and Technology* **2014**, *3*, 224–229.
